# Development of the symptomatic dermographism quality of life questionnaire

**DOI:** 10.1002/clt2.70038

**Published:** 2025-01-31

**Authors:** Melba Muñoz, Nicole Schoepke, Sabine Altrichter, Petra Staubach, Clara Geppert‐Steidl, Leslie Durner, Jonathan A. Bernstein, Marcus Maurer, Karsten Weller

**Affiliations:** ^1^ Institute of Allergology Charité ‐ Universitätsmedizin Berlin Corporate Member of Freie Universität Berlin and Humboldt‐Universität zu Berlin Mainz Germany; ^2^ Immunology and Allergology Fraunhofer Institute for Translational Medicine and Pharmacology ITMP Mainz Germany; ^3^ Department of Dermatology and Venerology Kepler University Hospital Linz Austria; ^4^ Department of Dermatology Johannes Gutenberg University Mainz Germany; ^5^ Division of Immunology and Allergy Department of Internal Medicine University of Cincinnati College of Medicine Cincinnati Ohio USA

**Keywords:** development, patient‐reported outcome, quality of life, symptomatic dermographism, urticaria

## Abstract

**Background:**

Symptomatic Dermographism (SD), also known as “urticaria factitia”, is the most common subtype of chronic inducible urticaria. Affected patients develop itch and strip‐shaped wheals that usually last for 30 min after minor stroking, rubbing, or scratching of the skin. The quality of life (QoL) of patients with SD is often strongly affected. However, a QoL instrument to properly assess this impairment is not yet available.

**Objective:**

The aim of this study was to develop the first disease‐specific patient reported outcome measure|patient reported outcome measures (PROM) to evaluate QoL impairment in SD patients, the Symptomatic Dermographism Quality of Life Questionnaire (SD‐QoL).

**Methods:**

SD‐QoL was developed following current guidelines for PROM development. We first generated a hypothetical conceptional framework of the SD‐QoL, followed by an item generation and an item selection/reduction phase.

**Results:**

During the item generation phase, 69 potential items of the SD‐QoL were generated by applying a combined approach consisting of literature review, expert input as well as semi‐structured interviews with affected patients. During the item selection phase, we reduced this long list of items to a final 13‐item set by means of impact analysis, inter‐item correlation, and additional criteria for item reduction, including an expert review for content (face) validity. Finally, a US‐American‐English version of the SD‐QoL was developed using a structured translation process.

**Conclusions and Clinical Relevance:**

The SD‐QoL is the first disease‐specific‐QoL instrument for SD with a recall period of 7 days that allows the assessment of QoL in SD patients. A subsequent validation study will determine its validity and reliability.

## 1 INTRODUCTION

Symptomatic dermographism (SD) also known as urticaria factitia or dermographic urticaria is the most common form of chronic inducible urticaria (CIndU).^1^ It affects 1%–5% of the general population and usually persists for many years before spontaneous remission.^2^, ^3^ In some cases, it has also been reported to be a familial disease.^4^, ^5^

SD is characterized by the recurrent appearance of itchy linear‐shaped wheals after skin exposure to shear forces, for example, light stroking, scratching, or rubbing that can last for 30 min to 1 h.^2^, ^6^ While a defining feature of SD is the appearance of wheals after scratching, most patients report that the scratching itself is a consequence of a generalized itch, which occurs spontaneously. Therefore, itch and the predisposition of the patient's skin to react with wheals after scratching or applying other shear forces are the main components of SD. These two components are not only important for the pathophysiological understanding of SD but also important for understanding and assessing the patient's disease burden.

Mast cell activation through autoreactive IgE antibodies with the subsequent release of histamine and other proinflammatory mediators are key pathophysiological events in all forms of urticaria.^7^, ^8^, ^9^, ^10^, ^11^, ^12^ A recent study showed that mast cells are essential for the development of SD since a single intravenous treatment with an anti‐KIT monoclonal antibody (mAb) barzolvolimab, depleted skin mast cells and completely abolished SD symptoms.^13^

Furthermore, the development of wheals in response to skin friction that characterizes SD was shown to be transferable by serum transfer experiments.^9^, ^10^ H_1_‐anthistamines are the first‐line and only licensed treatment option for SD. However, H_1_‐anthistamines often do not control SD symptoms, even when given continuously up to four times the recommended daily dose as recommended by the current guidelines.^14^ In some cases, skin moisturizers can help to reduce itch and alleviate the symptoms. Studies aiming a evaluating novel treatment options are urgently needed, but they are hampered by the lack of suitable outcome measures. Existing instruments in the field of urticaria, such as the Chronic Urticaria quality of life (QoL) Questionnaire (CU‐Q_2_oL),^15^ primarily developed for chronic spontaneous urticaria (CSU), do not consider the inducibility of signs and symptoms of CIndUs. Many SD patients report significant impairment of their QoL since their daily activities are affected by their symptoms.^1^, ^16^ Therefore, an instrument to assess QoL in SD patients is crucial to objectively evaluate the burden of the disease and response to therapy. The first disease specific health‐related quality of life (HRQoL) questionnaire for an inducible urticaria, the Cholinergic Urticaria QoL Questionnaire, is published,^17^ but disease‐specific questionnaires for all other CIndU subforms, including SD, are still not available. Generic HRQoL instruments might be otherwise applied to evaluate the impact of SD on the QoL of SD patients. However, these instruments are non‐specific and usually not sensitive enough to capture real disease burden, which is critical for patient assessment in clinical studies and in routine patient care.

To address this medical unmet need, we developed an SD‐specific patient reported outcome measure|patient reported outcome measures (PROM) that assesses QoL impairment in SD patients, called the symptomatic dermographism quality of Life Questionnaire (SD‐QoL).

## 2 METHODS

### 2.1 Patient sample and data acquisition

For the development of the SD‐QoL, ≥18‐year‐old patients were recruited at the outpatient clinic of the Department of Dermatology and Allergy, Charité ‐ Universitätsmedizin Berlin and at the Department of Dermatology of the University Medical Center in Mainz, Germany. Our study was approved by the local ethics committee and all patients provided written informed consent before participating in the study. This work consisted of three main phases: 1) assembly of an expert working group and generation of a conceptional framework of the SD‐QoL, 2) an item generation phase, and 3) an item reduction/selection phase. The development method was based on current recommendations for PROM^18^ as well as according to the Food and Drug Administration (FDA) guidance,^19^ European Medicines Agency recommendations^18^ and the recommendations of GA^2^LEN taskforce position paper.^20^

### 2.2 Expert group and conceptional framework

In a first step, an expert group was convened to take over several tasks: 1) to generate the conceptual framework of the SD‐QoL, 2) to provide input during the item generation phase, 3) to guide the item reduction process, 4) to review the item selection for content (face) validity, and 5) to define criterion measures (anchor instruments) to be applied during the validation study of the SD‐QoL. The expert group consisted of experts in the field of SD patient care in the field of clinical trials, and in the field of PROM development.

### 2.3 Item generation

In a second step, after the conceptional framework was outlined, potential questions (items) for the SD‐QoL were developed (unselected item long list). To this end, we performed exploratory semi‐structured interviews with SD patients (*n* = 10). The interviews asked about SD‐specific symptoms, their influence on daily activities, body care, physical and emotional well‐being as well as on social interactions. In addition, we performed a literature search on SD symptoms and their impact on affected patients as well as PROMs that have already been used in the field of SD. The expert group was asked to provide input on topics that needed to be addressed during the development of the SD‐QoL for example, finding the appropriate wording to define the items.

### 2.4 Item reduction and selection

In the third step, an item reduction was performed to delete items of low importance for the patients, to eliminate redundant items from the long list, and to ensure a good balance of topics regarding the content validity of the SD‐QoL and the corresponding burden. For the reduction phase, impact analysis and inter‐item‐correlation were performed. Moreover, the expert group defined the additional criteria for item selection and reviewed the selected items to determine the overall final item set.

### 2.5 Impact analysis

For the impact analysis and the inter‐item correlation, all generated items (unselected item long list) were handed out to 58 SD patients. All patients were asked to complete all items as well as to rate the importance of each item on a scale from 0 (not important) to 4 (very important). In addition, patients rated the content of each item in terms of its relevance to their SD symptoms within the last 12 months (answer option “yes” or “no”). Each item's “frequency” was then calculated as the percentage of patients who reported the relevance of the indicated item divided by 100. Subsequently, the impact scores were calculated by multiplying the mean of the “importance” by the “frequency” of each item.

### 2.6 Inter‐item correlation

An inter‐item correlation analysis was performed to identify redundant items by using Spearman rank correlation of all items selected during the impact analysis. The interpretation of the Spearman rank correlation coefficient was as follows: 0.5–0.699 good correlation, 0.7–0.899 strong correlation and ≥ 0.9 very strong correlation, which indicates redundancy of the respective items.

### 2.7 Additional criteria for the deletion of items

The most important criteria for the deletion of items were a low impact score in the impact analysis. However, additional criteria suggesting an item deletion were 1) high floor and ceiling values (≥30%) because this indicates a rather poor variability of responses, 2) a considerable number of missing responses (>5%), indicating a poor relevance or items that were difficult to understand, 3) strong (0.7–0.899) and very strong inter‐item‐correlation (≥0.9 correlation), reflecting redundancy. Moreover, the expert group deleted some items in favor of similar items with better properties and/or better content (face) validity.

### 2.8 Final formatting and cognitive debriefing of the symptomatic dermographism quality of life questionnaire

After the item reduction phase was completed, an instruction section (explaining how to complete this tool) was added to the final SD‐QoL. Subsequently, the final SD‐QoL was subjected to a cognitive debriefing with 10 SD patients to confirm the comprehensiveness and comprehensibility of the instruction section, all items and answer options.

### 2.9 Development of an American‐English version

After the final formatting of the SD‐QoL, a US American‐English version was developed. To this end, the German version of the SD‐QoL was translated to American‐English by two independent native US American‐English speakers that were bilingual in the source language German. Both forward translations were then reviewed, reconciled, and edited by a US American‐English native speaker and urticaria expert. Subsequently, a back‐translation was performed by a German native speaker, bilingual in American‐English, and this back‐translation was compared to the German source. After the US American‐English translation was regarded equivalent to the German version, it underwent cognitive debriefing with five US American‐English native speaking SD patients to test its comprehensiveness and comprehensibility.

### 2.10 Statistical analyses

All statistical analyses were performed using Statistical Package for Social Sciences (SPSS) (IBM SPSS Statistics Version 28.0.1; IBM Corporation).

## 3 RESULTS

### 3.1 Conceptional framework

The conceptional framework of the SD‐QoL was developed by the expert group. The target group of the SD‐QoL was defined as all adult patients with SD. The major functions of the SD‐QoL were specified as 1) the determination of the overall SD‐related QoL impairment as well as 2) the assessment of the pattern of impairment. Accordingly, the SD‐QoL was intended to have a total score (application option as an index instrument) as well as different QoL dimension scores (application option as a profile measure). An additional purpose of the SD‐QoL was defined to be the determination of changes of SD‐related QoL over time, for example, changes occurring during the change of treatments. The way of assessment was decided to be retrospective so that results are available instantly after administration and the SD‐QoL was intended as a self‐administered questionnaire (either on paper or electronically). Finally, it was decided that the SD‐QoL should contain content on the physical, social and emotional burden of SD as well as on the “functioning” of the patients in daily life.

## 4 SYMPTOMATIC DERMOGRAPHISM QUALITY OF LIFE QUESTIONNAIRE QUESTIONNAIRE DEVELOPMENT

### 4.1 Item generation

The combined approach of patient input, literature search, and expert input resulted in an unselected long list of 69 potential SD‐QoL items (Table 1). The semi‐structured interviews were performed with 10 SD patients (40% males and 60% females) with a median age of 35.5 years (33–40 years) and a median disease duration of 2 years (Table 2). Thirty‐four percent of patients in our study reported having another minor urticaria form. From these, 27% patients reported a CSU and 10% reported another CIndU subtype. Although, CSU and SD symptoms can be similar, the characteristic induction of strip‐shaped wheals after stroking of the skin in response to itch allows us to determine the impact of QoL due to SD‐specific symptoms. All SD patients referred that itch was the most frequent and the most relevant symptom and all patients indicated that they had an impaired QoL due to their disease. The mean urticaria control test (UCT) of our SD patients was nine points and the mean Dermatological life quality index (DLQI) was six points, which corresponds to poorly controlled disease and moderate impact on the QoL of these patients, respectively. Furthermore, 66% of patients in this study reported mild to moderate itch and 50% reported mild or no occurrence of hives. Therefore, our sample size represents all severity levels, from mild to moderate to severe SD cases. Based on the patient interviews and expert input, the item recall period was defined as 7 days. The item responses were configured as five‐point verbal rating scales. The answer options were designed to be clearly understood and to have similar intervals between the five options. Moreover, five response options were regarded as suitable to not bias the responses toward one direction and have also been proven to be a suitable approach in many other PROMs. The score range of all answer options was defined to be 0–4 points for all items to ensure equally weighted item scores, with low scores indicating responses associated with a mild QoL impairment and high item scores indicating responses associated with severe QoL impairment.

**TABLE 1:** Impact analysis.

Variable	Item	Importance	Frequency	Impact score
**Item_1**	**Itch (intensity)**	**3.05**	**0.95**	**2.89**
**Item_2**	**Wheals (intensity)**	**2.76**	**0.91**	**2.51**
**Item_3**	**Skin redness (intensity)**	**2.79**	**0.95**	**2.64**
Item_4	Skin pain (intensity)	1.57	0.50	0.79
Item_5	Skin burning (intensity)	1.98	0.65	1.29
Item_6	Skin sensation of heat (intensity)	1.81	0.65	1.17
**Item_7**	**Itch (frequency)**	**3.00**	**0.98**	**2.95**
**Item_8**	**Wheals (frequency)**	**2.60**	**0.86**	**2.24**
**Item_9**	**Skin redness (frequency)**	**2.68**	**0.91**	**2.44**
Item_10	Skin pain (frequency)	1.61	0.53	0.85
Item_11	Skin burning (frequency)	1.96	0.66	1.28
Item_12	Skin sensation of heat (frequency)	2.00	0.68	1.37
**Item_13**	**Limitation at school/study/work**	**2.57**	**0.80**	**2.06**
**Item_14**	**Limitation on family life**	**2.47**	**0.78**	**1.92**
Item_15	Limitation on housework	1.79	0.52	0.93
**Item_16**	**Limitation on leisure time**	**2.26**	**0.69**	**1.56**
**Item_17**	**Limitation on partnership**	**2.40**	**0.72**	**1.73**
Item_18	Limitation on social life	2.26	0.59	1.32
**Item_19**	**Limitation on sexual life**	**2.56**	**0.63**	**1.62**
**Item_20**	**Limitation on physical activities**	**2.29**	**0.67**	**1.54**
Item_21	Limitation on sports	2.12	0.60	1.26
**Item_22**	**Limitation on body care**	**2.40**	**0.71**	**1.70**
**Item_23**	**Limitation on choice of clothes**	**2.44**	**0.62**	**1.52**
Item_24	Limitation on choice of cosmetics	2.14	0.62	1.33
**Item_25**	**Impairment of sleep**	**2.19**	**0.70**	**1.54**
**Item_26**	**Unable to fall asleep**	**2.33**	**0.67**	**1.55**
Item_27	Unable to sleep through the night	2.19	0.60	1.31
Item_28	Waking up many times	2.22	0.60	1.33
Item_29	Tired during the day	2.07	0.53	1.09
Item_30	Difficulty to concentrate	2.22	0.63	1.40
Item_31	Mental performance	2.14	0.53	1.13
Item_32	Hiding skin lesions from others	2.07	0.57	1.18
Item_33	Avoiding public places	1.61	0.40	0.64
Item_34	Appearance	2.29	0.63	1.45
Item_35	Contact to others	2.00	0.47	0.95
Item_36	Feeling ashamed	2.12	0.49	1.04
Item_37	Feeling embarrased	2.12	0.58	1.23
Item_38	Fear from the reaction of other people	1.95	0.48	0.94
Item_39	Feeling of losing control of life	1.88	0.48	0.91
**Item_40**	**Feeling of no control over urticaria**	**2.53**	**0.67**	**1.69**
Item_41	Mood swings	2.18	0.57	1.24
**Item_42**	**Fear that disease might worsen**	**2.61**	**0.75**	**1.97**
**Item_43**	**Scratching frequency**	**3.18**	**0.93**	**2.96**
Item_44	Feeling of uncertainty	2.14	0.61	1.31
**Item_45**	**Anxious about drug side ‐ effects**	**2.34**	**0.71**	**1.67**
Item_46	Less achieved than aimed	1.79	0.44	0.79
Item_47	Preference to stay at home	1.74	0.39	0.67
Item_48	Limited overall performance	2.07	0.51	1.05
Item_49	Limitations on going out	1.78	0.43	0.77
Item_50	Having drug side ‐ effects	2.31	0.60	1.39
**Item_51**	**Emotionally burdened**	**2.67**	**0.72**	**1.92**
Item_52	Feeling nervous	2.14	0.60	1.29
Item_53	Feeling uneasy	2.23	0.64	1.42
Item_54	Feeling afraid	1.79	0.45	0.80
**Item_55**	**Feeling helpless**	**2.41**	**0.66**	**1.58**
**Item_56**	**Feeling at the mercy of the disease**	**2.33**	**0.67**	**1.57**
Item_57	Feeling powerless	2.11	0.68	1.44
**Item_58**	**Feeling frustrated**	**2.53**	**0.74**	**1.86**
Item_59	Feeling depressed	2.19	0.57	1.25
Item_60	Feeling discouraged	2.04	0.53	1.07
Item_61	Feeling downhearted	2.02	0.53	1.06
Item_62	Feeling sad	2.00	0.53	1.05
Item_63	Feeling tired	2.14	0.61	1.31
**Item_64**	**Feeling annoyed**	**2.67**	**0.81**	**2.15**
**Item_65**	**Feeling upset**	**2.28**	**0.67**	**1.52**
Item_66	Feeling angry	1.91	0.46	0.89
Item_67	Feelings aggressive	1.80	0.43	0.82
Item_68	Feeling stressed	2.32	0.63	1.46
Item_69	Feeling exhausted	2.10	0.57	1.20

*Note*: A combined approach of patient input, literature search, and expert input resulted in an unselected long list of 69 potential SD‐QoL items. An impact analysis was performed to select relevant and important items for the final SD‐QoL. Items with an impact score of ≥ 1.5 points are depicted in bold.

**TABLE 2:** Demographic characteristics.

SD patients	Semi‐structured interviews	Item selection and reduction phase	CD German
	*N* = 10	*N* = 58	*N* = 10
Male *N* (%)	4 (40%)	12 (21%)	4 (40%)
Female *N* (%)	6 (60%)	46 (79%)	6 (60%)
Age in years (median, IQR)	3.7 (33–40)	43 (31–50.5)	44.7 (30–61.2)
18–30 years (%)	2 (20%)	11 (19%)	3 (30%)
31–50 years (%)	7 (70%)	32 (56%)	3 (30%)
51–70 years (%)	1 (10%)	10 (18%)	4 (40%)
>70 years (%)	0	4 (7%)	0
SD duration in years (median, IQR)	2, (1–2)	4 (2–10)	1.9 (1.2–1.8)
<5 years (%)	10 (100%)	35 (65%)	9 (90%)
6–15 years (%)		9 (17%)	1 (10%)
16–25 years (%)		6 (11%)	
26–35 years (%)		2 (4%)	
>35 years (%)		2 (4%)	

*Note*: Age, gender and disease duration of SD patients participating in the item generation and reduction phase as well as in the cognitive debriefing interviews for german‐speaking participants.

Abbreviation: IQR, Inter Quartile Range.

### 4.2 Item selection

In the item selection phase, 46 females and 12 males were included (Table 2). Over 50% of the study population were between 31 and 50 years. Around 65% of the patients had a disease duration of 5 years or less. However, almost 20% of the patients reported a disease duration between 6 and 15 years (Table 2). Eighty percent of the patients were treated with antihistamines at the time of participation. The results of the impact analysis are shown in Table 1. Items were preselected when their impact score was ≥1.5 points (Table 1). This cut‐off value was recommended by the expert group since it allowed distinguishing between relevant and nonrelevant items as well as important and non‐important items. Further reduction was based on the inter‐item correlation analysis (Table 3), the additional criteria for item deletion and expert review for face validity (Table 4). Although, items 15 and 34 had an impact score < 1.5, the expert group decided to keep them due to their content (face) validity. In addition, items 13 and 15 as well as items 14 and 17 were consolidated into single items to ensure the face validity of the final SD‐QoL.

**TABLE 3:** Inter‐item correlation of items selected by the impact analysis.

Item	1	2	3	13	14	15	16	17	19	22	23	25	34	42	43	58	64
**1**	1	0.66	0.60	0.64	0.62	0.42	0.51	0.43	0.42	0.53	0.46	0.39	0.48	0.31	0.80	0.48	0.60
**2**	0.66	1	0.67	0.59	0.47	0.55	0.50	0.44	0.54	0.37	0.45	0.25	0.44	0.23	0.52	0.45	0.57
**3**	0.60	0.67	1	0.57	0.48	0.41	0.56	0.60	0.46	0.28	0.21	0.09	0.53	0.33	0.48	0.45	0.54
**13**	0.64	0.59	0.57	1	0.86	0.69	0.80	0.61	0.60	0.59	0.514	0.35	0.56	0.45	0.62	0.59	0.67
**14**	0.62	0.47	0.48	0.86	1	0.69	0.77	0.64	0.64	0.72	0.54	0.33	0.50	0.45	0.64	0.054	0.59
**15**	0.42	0.55	0.41	0.69	0.69	1	0.72	0.57	0.65	0.69	0.36	0.27	0.29	0.45	0.51	0.58	0.55
**16**	0.51	0.50	0.56	0.80	0.77	0.72	1	0.74	0.70	0.63	0.48	0.38	0.50	0.42	0.63	0.55	0.57
**17**	0.43	0.44	0.60	0.61	0.64	0.57	0.74	1	0.84	0.57	0.43	0.26	0.49	0.47	0.49	0.48	0.58
**19**	0.42	0.54	0.46	0.60	0.64	0.65	0.70	0.84	1	0.64	0.49	0.34	0.44	0.37	0.44	0.46	0.55
**22**	0.53	0.37	0.28	0.59	0.72	0.69	0.63	0.57	0.64	1	0.62	0.39	0.38	0.41	0.57	0.49	0.50
**23**	0.46	0.45	0.21	0.51	0.54	0.36	0.48	0.43	0.49	0.62	1	0.45	0.57	0.35	0.58	0.51	0.58
**25**	0.39	0.25	0.09	0.35	0.33	0.27	0.38	0.26	0.34	0.39	0.45	1	0.13	0.34	0.48	0.37	0.35
**34**	0.48	0.44	0.53	0.56	0.50	0.29	0.50	0.49	0.44	0.38	0.57	0.13	1	0.25	0.52	0.41	0.64
**42**	0.31	0.23	0.33	0.45	0.45	0.45	0.42	0.47	0.37	0.41	0.35	0.34	0.25	1	0.50	0.68	0.61
**43**	0.80	0.52	0.48	0.62	0.64	0.51	0.63	0.49	0.44	0.57	0.58	0.48	0.52	0.50	1	0.66	0.71
**58**	0.48	0.45	0.45	0.59	0.54	0.58	0.55	0.48	0.46	0.49	0.51	0.37	0.41	0.68	0.66	1	0.76
**64**	0.60	0.57	0.54	0.67	0.59	0.55	0.57	0.58	0.55	0.50	0.58	0.35	0.64	0.61	0.71	0.67	1

*Note*: An inter‐item correlation of preselected items after the impact analysis was performed during the reduction and selection phase Values represent the Spearman's rank correlation coefficient. Spearman's rank correlation coefficients of 0.7–0.899 indicate strong and 0.9–1 very strong correlations that may be interpreted as a marker of redundancy are highlighted in gray.

**TABLE 4:** Selection, reduction, or adjustment of items after impact analysis.

ID	Variable	Impact score	Missing value (%)	Floor value (%)	Ceiling value (%)	Inter‐item correlations	Expert consensus on selection
**Item_1**	**Itch (intensity)**	**2.9**	**<5%**	**5.3**	**7**	**7, **43	**IS > 1.5. Strong correlation with item 7 and 43, very low FV and CV. To be included in SD‐QoL**
**Item_2**	**Wheals (intensity)**	**2.5**	**<5%**	**24.6**	**1.8**	***8	**IS > 1.5. Redundant to item 8. Low FV and CV. To be included in SD‐QoL**
**Item_3**	**Skin redness (intensity)**	**2.6**	**<5%**	**6.9**	**6.9**	**9	**IS > 1.5. Strong correlation with item 9. Low FV and CV. To be included in SD‐QoL**
Item_7	Itch (frequency)	2.9	<5%	3.6	18.1	**1, **7	IS > 1.5. Strong correlation with item 1. Low FV and CV. Expert group decision: Not included in the SD‐QoL because of strong correlation and content redundancy (face validity) with item 1.
Item_8	Wheals (frequency)	2.2	<5%	26.3	8.8	***2	IS > 1.5. Redundant to item 2. Low FV and CV. Expert group decision: Not included in the SD‐QoL because of strong correlation and content redundancy (face validity) with item 2.
Item_9	Skin redness (frequency)	2.4	<5%	7	12.3	**3	IS > 1.5. Strong correlation with item 3. Low FV and CV. Expert group decision: Not included in the SD‐QoL because of strong correlation and content redundancy (face validity) with item 3.
**Item_13**	**Limitation at school/study/work**	**2.1**	**<5%**	**29.1**	**3.6**	**14, **16	**IS > 1.5. Strong correlation with item 14 and 16. Low FV and CV. To be included in SD‐QoL, consolidated with 15**
**Item_14**	**Limitation on family life**	**1.9**	**<5%**	**29.3**	**6.9**	**13, **16, *17	**IS > 1.5. Strong correlation with item 13, 16, 17. Low FV. To be included in SD‐QoL, consolidated with 17**
**Item_15**	**Limitation on housework**	**0.9**	**<5%**	**48.3**	**1.7**	**16	**IS < 1.5. Strong correlation with item 16. High FV, low CV. To be included in SD‐QoL, consolidated with 13.**
**Item_16**	**Limitation on leisure time**	**1.6**	**<5%**	**27.6**	**1.7**	**13, **14,**17	**IS > 1.5. Strong correlation with item 13, 14, 17, 19, 20. Low FV and CV. To be included in SD‐QoL**
						**19, **20	
**Item_17**	**Limitation on partnership**	**1.7**	**<5%**	**42.1**	**7**	**19, **20, *14, *51	**IS > 1.5. Strong correlation with item 14, 19, 20, 51. High FV, low CV. To be included in SD‐QoL, consolidated with 14**
Item_19	Limitation on sexual life	1.6	<5%	42.1	7	*13, *14, **16	IS > 1.5. Strong correlation with item 16, 17, 20. High FV, low CV. Expert group decision: Not included in the SD‐QoL because of strong correlation and content redundancy (face validity) with item 16 and 17.
						**17 *20	
Item_20	Limitation on physical activities	1.5	<5%	31	1.7	*13, *14, **16	IS > 1.5. Strong correlation with item 16, 17. High FV, low CV. Expert group decision: Not included in the SD‐QoL because of strong correlation and content redundancy (face validity) with item 16 and 17
						**17, *19, *22	
**Item_22**	**Limitation on body care**	**1.7**	**<5%**	**27.6**	**5.2**	**14	**IS > 1.5. Strong correlation with item 14. Low FV and CV. To be included in SD‐QoL**
**Item_23**	**Limitation on choice of clothes**	**1.5**	**<5%**	**27.6**	**5.2**	*22	**IS ≥ 1.5. Good correlation with item 22. Low FV and CV. To be included in SD‐QoL**
**Item_25**	**Impairment of sleep**	**1.5**	**<5%**	**46.6**	**3.4**	**26	**IS ≥ 1.5. Strong correlation with item 26. High FV, low CV. To be included in SD‐QoL**
Item_26	Unable to fall asleep	1.6	<5%	51.7	1.7	**25	IS > 1.5. Strong correlation with item 25. High FV, low CV. Expert group decision: Not included in the SD‐QoL because of strong correlation and content redundancy (face validity) with item 25.
**Item_34**	**Appearance**	**1.4**	**<5%**	**36.2**	**5.2**	*64, *68,	**IS < 1.5. Good correlation with item 64, 68. High FV, low CV. Expert group decision: To include in the SD‐QoL due to face validity.**
Item_40	Feeling of no control over urticaria	1.7	<5%	31	18	*42, *43, * 45, **51, **55, **58, **64 *65	IS > 1.5. Strong correlation with item 51, 55, 58, 64. High FV, low CV. Expert group decision: Not included in the SD‐QoL because of strong correlation and content redundancy (face validity) with item 64.
**Item_42**	**Fear that disease might worsen**	**2.0**	**<5%**	**24,6**	**12.3**	*40, *51, *58, *64	**IS > 1.5. Good correlation to item 40, 51, 58, 64. Low FV and CV. To be included in SD‐QoL.**
**Item_43**	**Scratching frequency**	**3.0**	**<5%**	**7**	**29.8**	*1, *7, *64	**IS > 1.5. Good correlation to item 1, 7, 64. Low FV and CV. To be included in SD‐QoL**
Item_45	Anxious about drug side ‐ effects	1.7	<5%	44.6	12.5	*51, *55, *56, 58*	IS > 1.5. Good correlation to 51, 55, 56, 58 items. High FV and low CV. Expert group decision: Not included in the SD‐QoL due to face validity.
Item_51	Emotionally burdened	1.9	<5%	25.9	12.1	*42, **55, **56, ** 58, **65, **64	IS > 1.5. Strong correlation with item 55, 56, 58, 64. Low FV and CV. Expert group decision: Not included in the SD‐QoL because of strong correlation and content redundancy (face validity) with item 64.
Item_55	Feeling helpless	1.6	<5%	41.4	19	**51, ***56, **58, **64	IS > 1.5. Redundant to 56, strong correlation to item 51, 58, 64. High FV and low CV. Expert group decision: Not included in the SD‐QoL because of strong correlation and content redundancy (face validity) with item 64.
Item_56	Feeling at the mercy of the disease	1.6	<5%	44.8	20.7	**51, ***55	IS > 1.5. Redundant to 55, strong correlation to item 51, 58, 64. High FV and low CV. Expert group decision: Not included in the SD‐QoL because of strong correlation and content redundancy (face validity) with item 64.
						**58,**64	
Item_58	Feeling frustrated	1.9	<5%	22.8	17.5	**51, **55, **64	IS > 1.5. Strong correlation with item 51, 55, 64. Low FV and CV. Expert group decision: Not included in the SD‐QoL because of strong correlation and content redundancy (face validity) with item 64.
**Item_64**	**Feeling annoyed**	**2.2**	**<5%**	**21.1**	**24.6**	*34, **51	**IS > 1.5. Strong correlation with item 51, 55, ,58, 65. Low FV and CV. To be included in SD‐QoL**
						**55, **58, **65	
Item_65	Feeling upset	1.5	<5%	31.6	14	**64	IS > 1.5. Strong correlation with item 64. High FV and CV. Expert group decision: Not included in the SD‐QoL because of strong correlation and content redundancy (face validity) with item 64.

*Note:* Items with impact score ≥ 1.5 were selected, except for items 15 and 34 due to face validity as well as items 19 and 58 due to redundancy and face validity. Items 13 and 15 as well as items 14 and 17 were consolidated into one item due to face validity. Additional items were excluded due to their inter‐item correlations and/or Visual Analogue Scale/Verbal Rating Scale QoL correlations. Some items with an impact score > 1.5 were excluded due to very strong and strong inter‐item correlations (redundancy) (e.g. items 7, 8, 9, 20, 26, 40, 45, 51, 55, 56 and 65). High floor values (≥30%) of items 20, 26, 40, 45, 55, 56 and 65 were also considered in the selection phase. After the expert panel discussion, a final total number of 13 items were selected for the validation phase. IS (impact score), floor values (FV). Items included in the final SD‐QoL are depicted in bold.

*good correlation (0.5–0.699), **strong correlation (0.7–0.899), ***very strong correlation (≥0.9).

In order to confirm that the item selection was valid for both genders, we also performed the impact analysis on females and males separately. Both genders had comparable impact scores, suggesting that there were no major gender differences. However, there was a general tendency that females provided higher relevance scores.

### 4.3 Final formatting of the symptomatic dermographism quality of life questionnaire and cognitive debriefing

The item development and item reduction process resulted in a total of 13‐items. During the final formatting of the SD‐QoL, an instruction section was added. The final SD‐QoL instrument is shown in Figure 1. In the subsequent cognitive debriefing with 10 SD patients (6 females; median age: 42 years), with a median disease duration of 18 months, no difficulties in understanding the instructions, the single items, or the answer options were reported. In addition, there were no requests for further changes to the questionnaire.

FIGURE 1German version of the symptomatic dermographism quality of Life Questionnaire (SD‐QoL). A final 13‐item set with instructions and answer options configured as 5‐point verbal rating scale.

**Figure:**
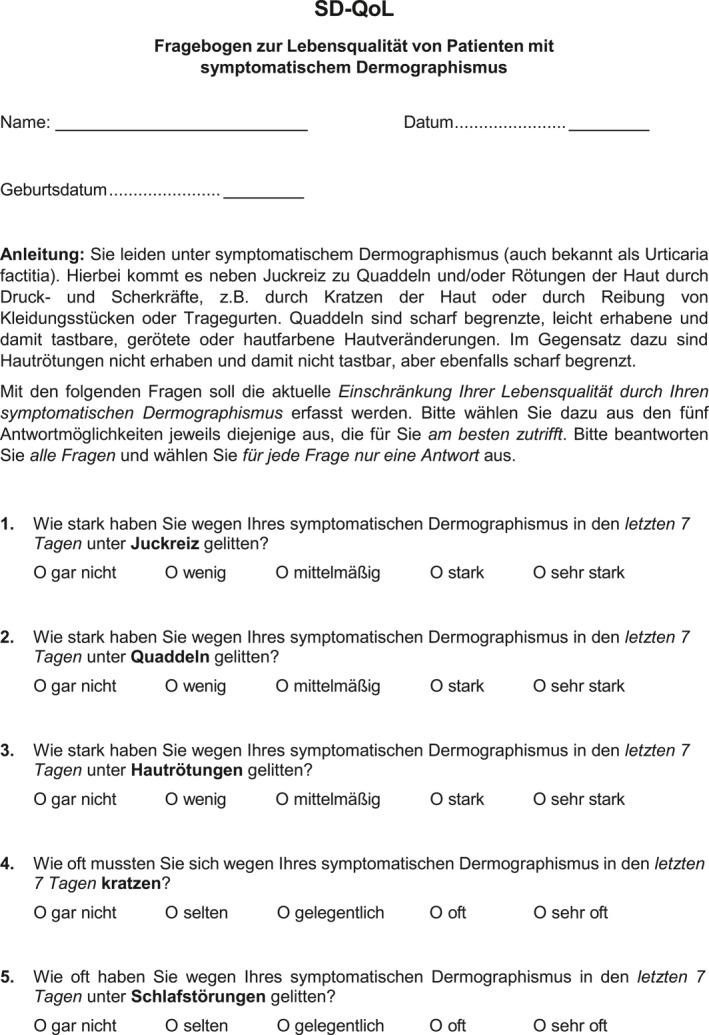

**Figure:**
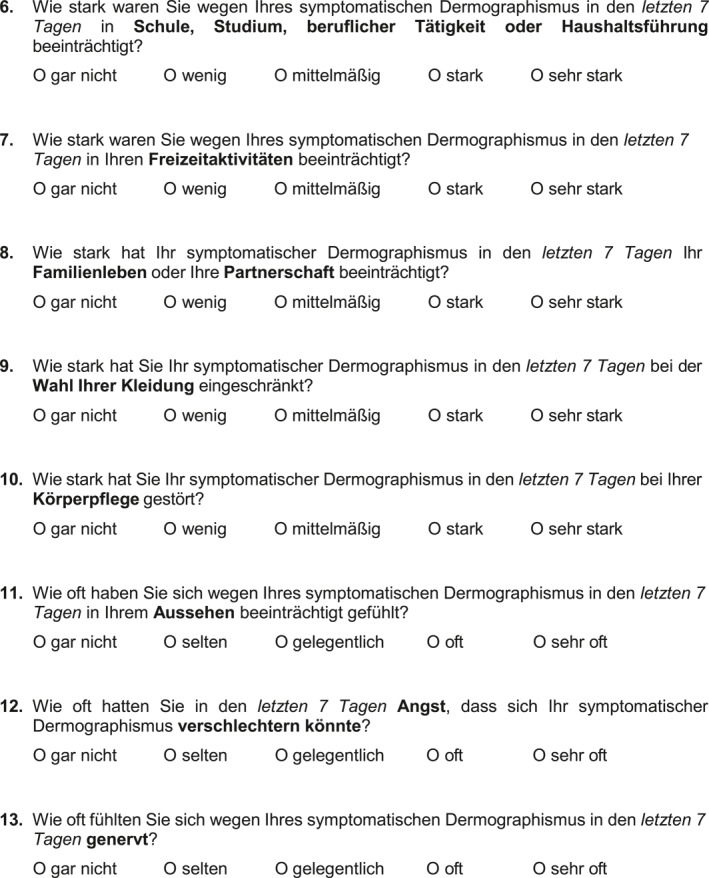

### 4.4 Development of an American‐English version

In addition to the original German version of the SD‐QoL, we developed a US American‐English version (Figure 2) as described in the methods section. Cognitive debriefing of the US American‐English version was performed with 5 US American‐English native speaking SD patients (3 female, 2 male, median age: 35.6 years) who confirmed that the US American‐English version is clear to understand.

FIGURE 2English version of the symptomatic dermographism quality of Life Questionnaire (SD‐QoL). A final 13‐item set with instructions and answer options configured as 5‐point verbal rating scale.

**Figure:**
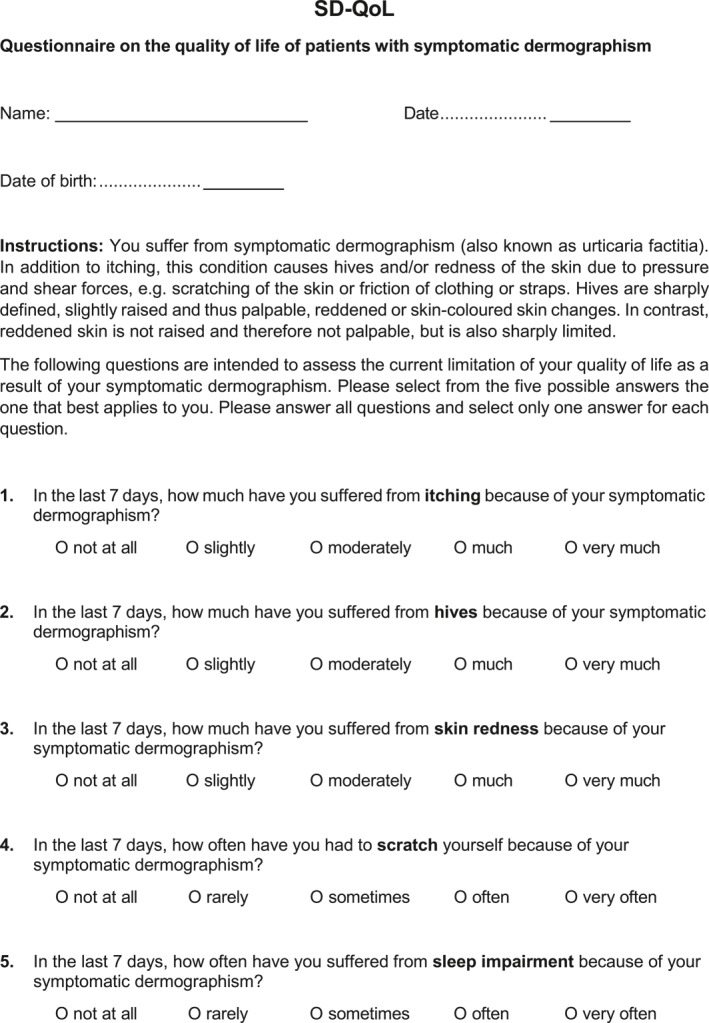

**Figure:**
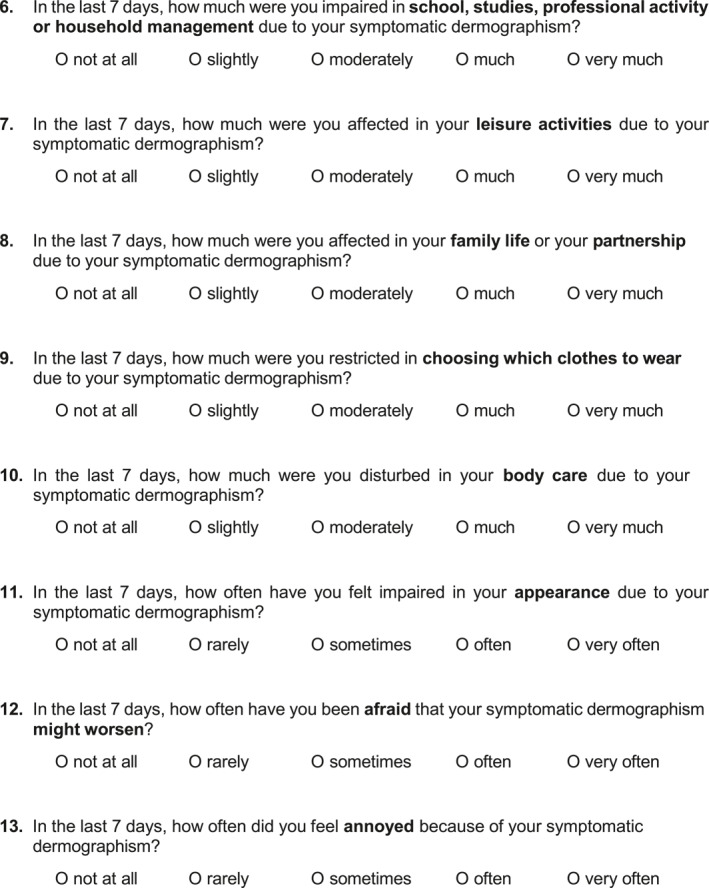

## 5 DISCUSSION

Symptomatic dermographism is the most common type of CIndU, characterized by a long duration and frequently by a strong impairment in QoL.^1^ QoL scores are important instruments used in the daily clinical care as well as in clinical trials to assess the impact of diseases on patient's performance, physical, emotional and social well‐being.^21^ As of now, there is no specific tool available to evaluate the QoL in SD patients. Here, we report the development of such an instrument, the first PROM to retrospectively assess the QoL in SD patients, the SD‐QoL. Validation and reliability of the SD‐QoL will be conducted in a separate study.

After the development of a conceptual framework for the SD‐QoL, 69 potential items were generated using a combined method of patient's interviews, literature search, and expert input. In the subsequent item selection and reduction phase, the results of the impact analysis were the main criterion for item deletion. This ensured that the patient perspective was rated highest in the development process. Additional criteria for item selection included an inter‐item correlation, missing values, floor and ceiling effects and an expert review for face validity. This combined approach allowed us to select the most important items and reduce the final item set to 13 questions, keeping a low respondent burden.

Although the final SD‐QoL covers some similar areas as the Chronic Urticaria QoL Questionnaire (CU‐Q_2_oL),^15^ the SD‐QoL does not harbor any item related to the treatment and the recall period was seven instead of 14 days. CU‐Q_2_oL is a validated PROM; however, it does not assess trigger exposure or avoidance, which is crucial for SD patients' symptom burden and its impact on QoL. Therefore, the CU‐Q_2_oL is not well suited to assess QoL in SD patients. In addition, comparable QoL scores that also evaluate the impairment of QoL, such as the CU‐Q2oL,^15^ AE‐QoL^22^ and CholU‐QoL^17^, also used a five‐point Likert scale and were therefore prototypes for the development of the SD‐QoL.

Antihistamines are the only licensed treatment for SD as well as other chronic inducible urticarias.^14^ However, many SD patients taking antihistamines do not achieve sufficient control of their symptoms.^6^ Moreover, moisturization of the skin can be used to mitigate the itch and reduce the itch‐scratch cycle. Clinical trials that assess novel therapies for SD patients are needed. For this, validated PROMs that evaluate and compare the response of different therapies in clinical studies are mandatory. SD‐QoL provides a standardized way to objectively assess patients' disease‐related QoL impairment in routine clinical care and will be an important instrument in clinical trials. In addition, the SD‐QoL is easy to fill out during regular outpatient visits, taking approximately less than 5 min to complete.

The strengths of our study include that SD patients with a broad range of disease durations, disease activities, different ages, both genders and in two independent centers were included in our analysis and that the patients' input was the major source during the SD‐QoL development. As expected, a large proportion of the study population was female since higher prevalence of females is observed in chronic urticaria. Moreover, SD‐QoL exhibited high comprehensibility, as demonstrated during the cognitive debriefing. Another strength of this work is that it was accompanied by an experienced expert group with knowledge in the field of SD as well as PROM development.^17^, ^22^, ^23^, ^24^ Limitations of our study include: (i) the relatively small sample size, (ii) only adults were included, (iii) since the participating centers were tertiary care centers, a selection bias might be present with participation of strongly affected patients. However, our sample size represents all severity levels from mild, moderate to severe SD cases based on the UCT and the DLQI scores. Nonetheless, the FDA guidance document points out that the number of patients is not as critical as interview quality and patient diversity included in the study.^19^ Lastly, content (face) validity review could encounter a certain degree of bias since this process depends on the opinion of the expert working group.

In conclusion, the final 13‐item SD‐QoL is the first PROM to specifically assess QoL in SD patients. Its retrospective approach and user‐friendliness ensure that it is an easy tool for daily patient care as well as clinical trials. The current ongoing validation study will provide important additional insights into the validity and reliability of the SD‐QoL in the near future.

## CONFLICT OF INTEREST STATEMENT

The authors declare no conflicts of interest.

## Data Availability

Data available on request from the authors.
